# The Assessment of Isometric, Dynamic, and Sports-Specific Upper-Body Strength in Male and Female Competitive Surfers

**DOI:** 10.3390/sports6020053

**Published:** 2018-06-05

**Authors:** Joanna Parsonage, Josh Secomb, Rebecca Dowse, Brendon Ferrier, Jeremy Sheppard, Sophia Nimphius

**Affiliations:** 1 Surfing Australia High Performance Centre, Casuarina Beach 2487, Australia; RDOWSE@our.ecu.edu.au; 2 Centre for Exercise and Sports Science Research, School of Medical and Health Sciences, Edith Cowan University, Joondalup 6027, Australia; joshsecomb37@gmail.com (J.S.); B.Ferrier@napier.ac.uk (B.F.); jsheppard@csipacific.ca (J.S.); s.nimphius@ecu.edu.au (S.N.); 3 Canadian Sports Institute-Pacific, Whistler VON 1BO, Canada; 4 Queensland Academy of Sport, Nathan 4111, Australia; 5 School of Applied Science, Edinburgh Napier University, Edinburgh EH11 4BN, Scotland, UK

**Keywords:** assessment, skill, performance, pop-up, gender

## Abstract

The primary purpose of this study was to investigate gender differences in the dynamic strength index (DSI): an assessment of upper-body dynamic strength relative to maximal isometric strength. The secondary purpose was to investigate gender differences in the dynamic skill deficit (DSD): an assessment of sports-specific dynamic strength relative to maximal isometric strength, and its association with a sports-specific performance measure in surfers. Nine male (age = 30.3 ± 7.3 yrs) and eight female (age = 25.5 ± 5.2 yrs) surfers undertook three upper-body assessments: isometric push-up, dynamic push-up, and a force plate pop-up to determine the DSI and DSD. The performance measure of time taken to pop-up (TTP) was recorded. No gender differences for the DSI (*d* = 0.48, *p* = 0.33) or DSD (*d* = 0.69, *p* = 0.32) were observed. Normalized peak force (PF) of the isometric push-up, dynamic push-up, and force plate pop-up were significantly greater in males (*p* ≤ 0.05), with males recording significantly quicker TTP (*d* = 1.35, *p* < 0.05). The results suggest that male and female surfers apply a similar proportion of their maximal strength in sports-specific movements. However, greater normalized isometric and dynamic strength in males resulted in greater sports-specific PF application and a faster TTP. It would appear favorable that female surfers improve their maximal strength to facilitate sports-specific pop-up performance.

## 1. Introduction

The sport of surfing incorporates three crucial phases: paddling, pop-up, and the wave-ride [1]. The pop-up phase of surfing is characterized by the change from a prone paddling position to a surf-specific standing position in one dynamic movement [2]. During this transition, a surfer is required to move ~75% of their body weight in less than a second [3]. Therefore, upper-body strength is a key physiological capacity to assist in the execution of a fast and effective pop-up.

Isometric and dynamic testing protocols have been previously implemented to assess upper-body strength and power qualities in male and female athletes [4]. To determine an athlete’s dynamic force capabilities in relation to their maximum isometric strength, comparisons between isometric and dynamic strength measures have been made [5]. Sports scientists refer to this as the dynamic strength index (DSI). The DSI is expressed as a ratio of dynamic peak force (PF) to isometric PF, and has been shown to be highly reliable in assessing strength qualities in both the lower [6,7] and upper-body [5].

The DSI for the upper-body has previously been calculated using the isometric bench press and ballistic bench throw testing protocols [5]. Young and colleagues [5] concluded that the DSI was a reliable and valid means of assessing upper-body maximal strength capabilities and was sensitive enough to detect training-induced changes in male athletes (ICC = 0.93, CV = 3.5%). At present, there is a lack of research examining an upper-body DSI for a female athlete cohort. It has been reported that males demonstrate significantly greater normalized maximal upper-body strength when executing a one repetition maximum chest press [8]. Similarly, research exploring gender differences in upper-body dynamic strength identified that males possessed significantly higher upper-body dynamic strength than females, even when fat-free mass was controlled for [9]. Thus, the DSI may provide strength and conditioning practitioners with a greater insight into the specific upper-body strength qualities of male and female surfers and subsequently guide targeted training interventions.

The dynamic skill deficit (DSD) is aimed at assessing sports-specific dynamic strength capabilities in relation to maximal isometric strength. It has previously been supported that strength is associated with sports-specific skill. Examples of this include greater maximal strength associated with faster throwing velocity in handball players [10], greater peak power output associated with a higher vertical jump height in elite volleyball players [11], and greater normalized strength being highly associated with speed and change of direction in softball players [12]. In relation to surfing, Parsonage et al. [13] previously investigated normalized PF application during an isometric (IPU) and dynamic push-up (DPU), and a surf-specific pop-up (FP POP), with the performance measure of time to pop-up recorded (TTP). It was reported that stronger surfers produced greater normalized isometric and dynamic upper-body strength with large magnitude difference in PF applied in the FP POP (*d* = 0.80, *p* = 0.08) and a quicker TTP (*d* = 0.85, *p* = 0.07). Based on these findings, it may be beneficial to examine the application of maximal isometric strength within the context of a sports-specific movement.

The primary purpose of this study was to investigate the gender differences in the DSI as a means of assessing a surfer’s upper-body dynamic strength qualities in relation to their maximal isometric strength. It was hypothesized that there will be a significant difference in DSI between male and female surfers coupled with significantly different normalized upper-body isometric and dynamic strength. The secondary purpose of the study was to investigate the concept of a DSD, and specifically gender differences, in sports-specific dynamic strength capabilities in relation to maximal isometric strength and its association with a sports-specific performance measure. It was hypothesized that there will be a significant difference in DSD between genders, with a significant difference in sports-specific dynamic strength capabilities and surf-specific TTP noted.

## 2. Materials and Methods

### 2.1. Participants

Eighteen competitive surfers (28.1 ± 6.5 yrs, 69.6 ± 10.4 kg, 172.5 ± 6.7 cm), nine male and nine female, were recruited for the current study. However, a single female participant was excluded from the study due to maximal effort not being achieved in the isometric upper-body assessment. Physical characteristics of the seventeen surfers are presented in Table 1. All participants had surfed for a minimum of 10 years and surfed on average more than three times a week. Participants were free of any musculoskeletal injuries or medical conditions contraindicative of performing maximal exercise. All participants were provided an information letter explaining the benefits and risks of participation, and participants provided written informed consent prior to participation. Written informed assent was also obtained from a parent/guardian if the participant was under 18 years of age. Edith Cowan University Human Research Ethics Committee approved the research and all procedures (13013). It must be acknowledged that a portion of the raw data analyzed in the current study has previously been published [13]. However, the current research questions are unique in their purpose, method of analysis, and subsequent findings.

### 2.2. Anthropometry

Stature was measured to the nearest 0.01 m using a wall-mounted stadiometer (Aaxis SM height measure 2m, Blacktown, NSW, Australia), while body mass was recorded to the nearest 0.01 kg using a calibrated electronic scale. Four skinfold sites were measured (bicep, tricep, subscapular, supra-iliac) by an International Society for the Advancement of Kinanthropometry accredited practitioner using harpen skinfold calipers (British indicator, Hertfordshire, UK). The practitioner had a typical error of measurement (TEM) of 1.12–1.70%.

### 2.3. Study Design

Participants completed three upper-body strength assessments: isometric push-up (IPU), dynamic push-up (DPU), and force plate pop-up (FP POP) [13]. Participants were advised to refrain from any vigorous training 48 hours prior to testing on both days. The same standardized warm-up was undertaken by all participants, consisting of five repetitions of inclined push-ups performed at 60 cm, 45 cm, and 30 cm in a descending order.

The three upper-body strength assessments were performed on a force platform (400 Series Performance Force Plate, Fitness Technology, Adelaide, Australia) sampling at 600 Hz. The force platform was interfaced with computer software (Ballistic Measurement System, Fitness Technology, Adelaide, Australia) for measurement of force-time characteristics. The force plate was calibrated prior to each data collection, using a two-point calibration for a fitted regression as per the manufacturer instructions. All upper-body strength assessments (IPU, DPU, and FP POP) have previously demonstrated high between day reliability by the current researchers (ICC = 0.90–0.96, CV% = 4.4–5.0) [13].

The normalization of PF was carried out in accordance with previous research [13]. Participants were required to lay prone, with the chest placed on a yoga block and hands placed at approximately 100% of biacromial width. Holding this position for a period of five seconds, the average PF over three second was used to normalize for body weight. The IPU required participants to adopt a prone lying position, making sure a straight line between the torso and lower-body was maintained, while a modified pull-up belt was placed over their thoracic spine. Ensuring an elbow flexion of 100° was maintained, participants were instructed to “push the ground away as hard as possible” for a period of five seconds. The DPU was initiated by an entirely concentric contraction from a prone lying position. Participants were instructed to explosively push-up, extending their elbows from a fully flexed to a fully extended position, prior to them making contact with the force plate again. The FP POP required participants to pop-up from a prone lying position, to their surf-specific stance in one explosive concentric movement. For all assessments, the best trial, as determined by the highest normalized PF was used for subsequent analysis. The DSI was expressed as a ratio of normalized dynamic PF: isometric PF. The DSD was expressed as a ratio of normalized force plate pop-up PF: isometric PF.

In addition, the pop–up phase of the FP POP was analyzed from the time at which the participant’s chest left the force plate to the time of front foot contact. This was referred to as time to pop-up (TTP). Video footage was recorded using a GoPro (HERO3 Silver Edition HD3.02.03.00, CA, USA) sampling at a rate of 100 frames per second.

### 2.4. Statistical Analysis

All data are presented as mean ± standard deviation. Normality of data was assessed using the Shapiro-Wilk statistic, and homogeneity of variance between males and females was verified with the Levene’s test of equality. An independent sample t-test was conducted to determine if a significant difference in DSI and DSD between male and female surfers. Furthermore, independent sample t-tests were also conducted to determine whether there was a significant difference in normalized isometric and dynamic strength measures (IPU, DPU, and FP POP), as well as TTP. Sequential Bonferroni correction for multiple comparisons was applied [14]. Magnitude of effect was classified as follows; <0.2 (trivial), >0.2 (small), >0.5 (medium), and >0.8 (large) [15].

Pearson product moment correlations were conducted to assess the association between normalized isometric and dynamic upper-body strength measures and both the DSI and DSD for male and female surfers. In order to demonstrate explained variance, the coefficient of determination (*r*^2^) was calculated. Furthermore, Pearson product moment correlations were conducted to assess the association between DSD and TTP. A fisher’s *r*-Z transformation was performed to examine if there was a significant difference in normalized isometric strength and DSI and DSD associations between male and female surfers. All statistical analyses were performed using PRISM (Version 7.0b; GraphPad Software, Inc., La Jolla, CA, USA), and significance was set at *p* ≤ 0.05.

## 3. Results

### 3.1. Descriptive Characteristics for Male and Female Surfers

The mean ± standard deviations of the descriptive characteristics for male and female surfers are presented in Table 1.

### 3.2. Dynamic Strength Index (DSI)

**Figure 1:**
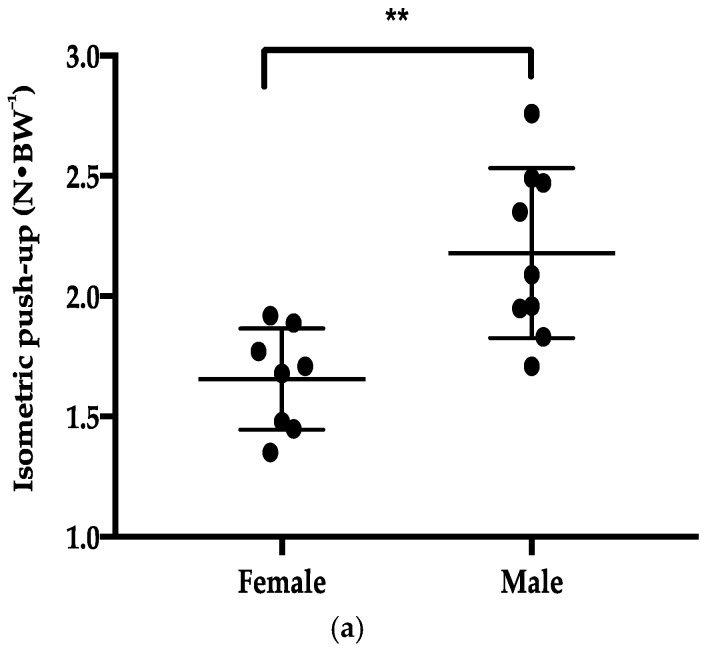

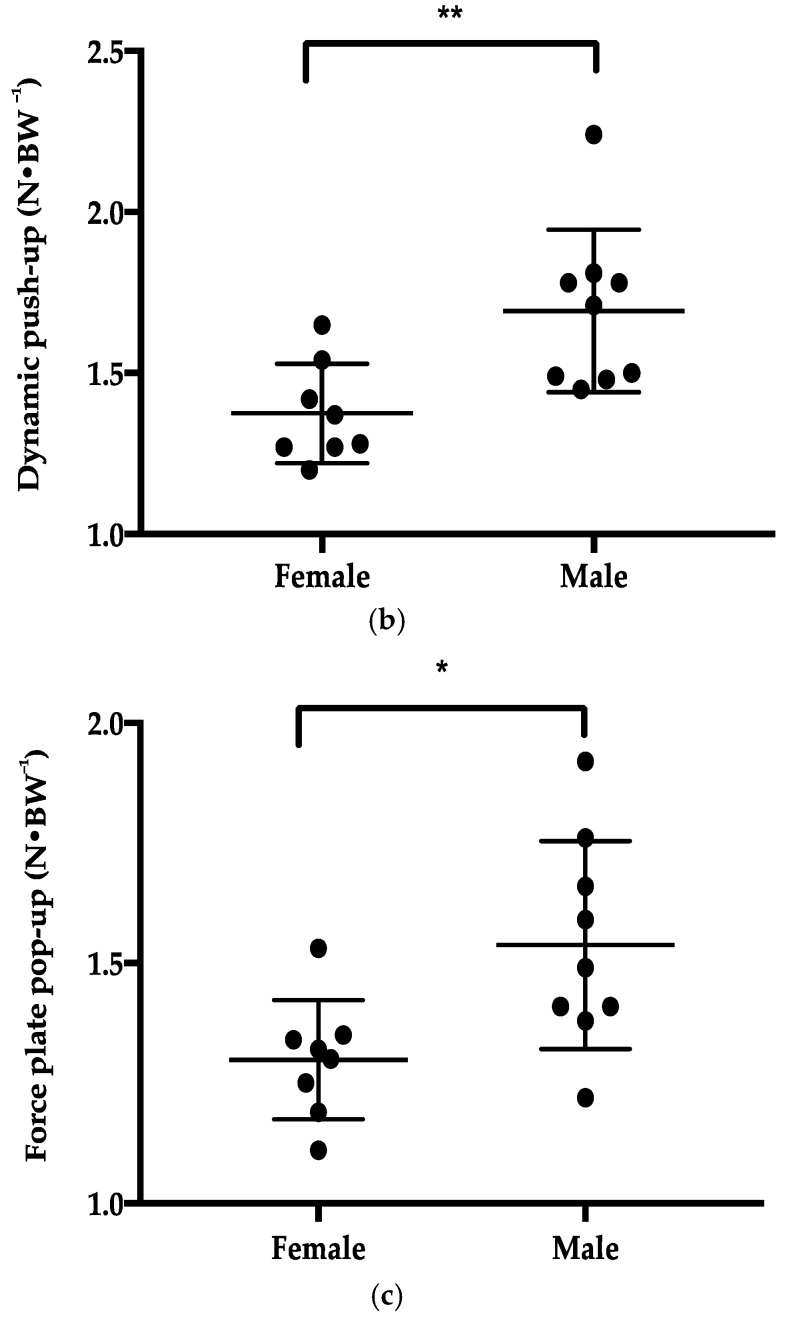
Reporting mean ± standard deviations of normalized peak force for male (n = 9) and female surfers (n = 8), for the three upper-body strength assessments: (**a**) Isometric push-up, (**b**) Dynamic push-up; (**c**) Force plate pop-up. Significance at * *p ≤* 0.05 ** *p ≤* 0.01.

The DSI showed a non-significant small magnitude difference (*d* = 0.48, *p* = 0.33) between male and female surfers (Table 2). However, there was a significant difference in normalized IPU PF (*d* = 1.33, *p* = 0.01) (Figure 1a) and normalized DPU PF (*d* = 1.21, *p* = 0.01) (Figure 1b) between male and female surfers.

### 3.3. Dynamic Skill Deficit (DSD) and Force Plate Time to Pop-Up (FP TTP)

The DSD showed a non-significant, moderate magnitude difference (*d* = 0.69, *p* = 0.32) between male and female surfers (Table 2). Male surfers produced significantly greater (*d* = 1.12, *p* < 0.05) normalized PF production in the FP POP (Figure 1c) and recorded significantly quicker (*d* = 1.35, *p* < 0.05) TTP (0.56 ± 0.06 s) compared to female surfers (0.68 ± 0.07 s).

### 3.4. Correlation Analysis

**Figure 2:**
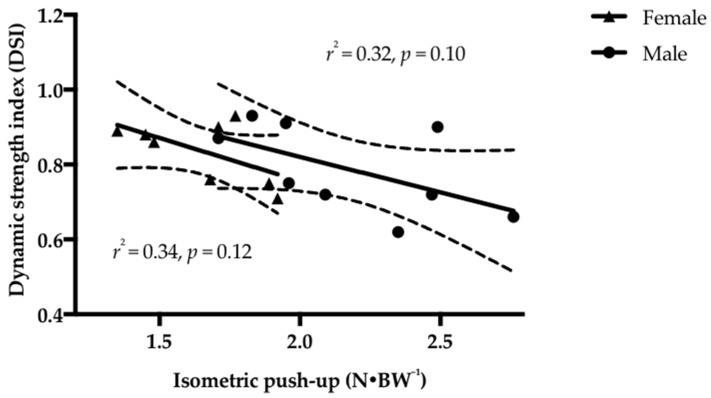
Linear regression with 95% confidence intervals and explained variance (*r*^2^) between isometric push-up (IPU) and dynamic strength index (DSI) in female and male surfers. No significance at *p ≤* 0.05.

**Figure 3:**
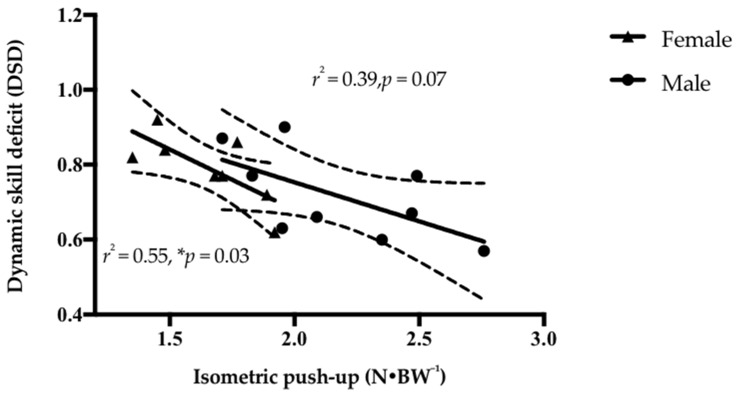
Linear regression with 95% confidence bands and explained variance (*r*^2^) between isometric push-up (IPU) and dynamic skill deficit (DSD) in female and male surfers. Significance at * *p ≤* 0.05.

Inverse associations between IPU and DSI occurred in male (*r* = −0.57, *p* = 0.10, 95% CI = −0.89, 0.14) and female surfers (*r* = −0.59, *p* = 0.12, 95% CI = −0.91, −0.20) but were not significant (Figure 2). Significant inverse associations were found between IPU and DSD in females (*r* = −0.73, *p* = 0.03, 95% CI = −0.95, −0.06) (Figure 3). No significant associations were reported between DSD and TTP in either male (*r* = 0.58, *p* = 0.10, 95% CI = −0.13, 0.89) or female surfers (*r* = 0.01, *p* = 0.99, 95% CI = −0.70, 0.71). However, the strength of the association was moderate for males while mild in females [15].

The fisher’s *r*-Z transformation performed on all associations found no significant differences in correlation coefficients between male and female surfers.

## 4. Discussion

The primary purpose of this study was to investigate the gender differences in the DSI as a means of assessing a surfer’s upper-body dynamic strength qualities in relation to their maximal isometric strength. The secondary purpose of the study was to investigate the concept of a DSD aimed at assessing gender differences in sports-specific dynamic strength capabilities in relation to maximal isometric strength, and its association with a sports-specific performance measure. No significant difference in either the DSI or DSD was found between male and female surfers. However, normalized PF in the IPU, DPU, and FP POP were significantly greater in males (*p* ≤ 0.05), coupled with a significantly quicker TTP (*p* < 0.05). Together, these data suggest that female and male surfers do not differ in the ability to apply their maximal isometric strength in a sports-specific movement (pop-up). However, the greater normalized isometric and dynamic PF application by male surfers appear to enable them to perform the surf-specific pop-up faster, which is a critical component of surfing performance.

The DSI of male surfers in the current study was 0.79 ± 0.12. It has been suggested that, for comparisons between PF data attained during an isometric bench press and a ballistic bench throw, a DSI ≤ 0.75 indicates relatively balanced maximal and dynamic strength [5]. Although the ratio in the current study is slightly higher than the threshold that Young et al. propose [5], the minor differences could be attributed to differences in methodology, as the present study did not allow for a countermovement as part of the DPU, in contrast to Young’s ballistic bench throw. This rationale has recently been supported by Comfort et al. [16], who highlighted that although the DSI calculated using a countermovement jump and squat jump was similar, the DSI calculated using the countermovement jump provided a more reliable and less variable measurement. As such, this study has begun to provide population specific upper-body ratios for surfers.

The lack of significant difference in DSI between genders indicates they apply similar relative magnitude force in the restricted time of the movement, similar to prior findings, albeit in the lower-body, demonstrating that when rate of force development is reported relative to maximum strength there is no significant difference between males and females [17]. A higher DSI has typically been interpreted as a need to increase maximum strength [5,18]. Such a conclusion must be made in context to maximum strength, therefore the suggestion to increase strength is supported by the combination of a high DSI and significantly lower normalized IPU PF in female surfers. The DSI in female surfers is a small magnitude higher than the upper-body baseline DSI values reported by Young et al. [5] in active males and the male surfers of the current study. Therefore, it may be of benefit for female surfers to firstly address their lower maximal strength levels, in order to facilitate PF application during a dynamic movement [19]. Although non-significant, the moderate magnitude inverse relationship (*r* = −0.59, *p* = 0.12) between DSI and normalized isometric PF suggests as a female’s strength increases the DSI declines, indicating that at a point when it drops below the aforementioned threshold that the DSI may then be at a level where it is appropriate to emphasize rate of force development.

The second purpose of the study was to investigate the concept of a DSD aimed at assessing gender differences in sports-specific dynamic strength capabilities in relation to maximal isometric strength, and its association with a sports-specific performance measure. The DSD was not significantly different between genders, suggesting that both male and female surfers apply a similar proportion of their maximal strength in a sports-specific pop-up. Despite the similarity in the DSD, males produced significantly greater normalized PF production in the sports-specific FP POP (*d* = 1.12, *p* = 0.04), coupled with a significantly quicker TTP (*d* = 1.35, *p* = 0.01). The aforementioned findings may indicate that the faster TTP by male surfers may not just be attributed to the greater normalized isometric and dynamic upper-body force application. Previous research has documented a significant disparity is muscle-mass distribution between genders, with 44% less upper-body muscle mass reported in females [20]. The combination of a greater force application, in addition to greater upper-body muscle mass in male surfers, may enable them to utilize their force application in a manner that maximizes their ability to draw their legs underneath them for a quicker TTP.

A quicker pop-up would allow a surfer to transfer from a prone paddling position to a surf-specific standing position faster, enabling them to commence the wave ride earlier. Previous literature has presented the notion that sports-specific skill is associated with an individual’s physical capacity [21,22]. The significant inverse associations reported between the DSD and normalized IPU PF in females (*r* = −0.73, *p* = 0.03) suggests maximal isometric strength may underpin dynamic strength capabilities in a sports-specific context. Marques et al. [23], reported than a 12-week resistance training programme resulted in a significant increase in both four-repetition maximum bench press and overhead medicine ball throw distance in elite female volleyball players. They concluded that a structured resistance training program including both maximal strength and plyometric exercise improved upper-body strength and power, thus facilitating volleyball-related performance. The application of the aforementioned findings to a female surfer population may be favorable in facilitating both strength capabilities and sports-specific performance.

The difference in sports-specific TTP between male and female surfers may also be attributed to better sports-specific motor skills of the males in the current study. Surfing is a unique sport in that males and females train in the same environment, competing for the same waves on a daily basis. Previous research has reported females to exhibit slower sprint paddle speed [24], compromising their ability to catch waves and subsequently limiting the number of pop-ups they perform. Therefore, although both male and female surfers may have similar sport-specific training ages, there could be a large discrepancy in the opportunities to practice the pop-up between genders. However, a limitation of the current study is that it did not document sport-specific training age, which may be advantageous in highlighting potential motor skill contributions. Furthermore, the current study had a small sample size to compare competitive male (n = 9) and female (n = 8) surfers. Although all of the participants were representative of a competitive cohort, the small population pool of such surfers may warrant future research across both recreational and competitive levels.

Although the current study highlights there are some significant gender differences in normalized isometric, dynamic, and sports-specific strength, the variability within genders also needs to be noted. All three upper-body strength measures (IPU, DPU, and FP POP) exhibited a large amount of overlap in performance between male and female surfers (see Figure 1, Figure 2 and Figure 3). Potential explanations for this could be multifactorial, including strength-training age [25], sociocultural factors [26], and self-objectification [27]. Therefore, it should be acknowledged that gender might not always be a determining or decision-making factor on its own in strength and sports-specific performance.

This is the first study to report gender differences in maximal strength using an isometric push-up. The IPU has previously been shown to be a reliable tool in the assessment of upper-body maximal strength [13]. Its implementation as a performance test may be favorable due to the familiar motor pattern recruited and the limited requirement of gym equipment. This is also the first study to investigate an upper-body DSI in female athletes, regardless of the protocol implemented. Future research should examine the effect of a maximal strength training intervention on the DSI and DSD, as well as the sport-specific performance measure of TTP in female surfers.

## 5. Conclusions

The DSI and DSD may be used as diagnostic tools in the assessment of upper-body strength qualities in male and female surfers. The DSI and DSD were not significantly different between genders. However, the isometric and dynamic strength qualities underpinning these ratios were significantly greater in male surfers, facilitating sports-specific performance (TTP). It would therefore appear favorable that female surfers focused on improving their maximal strength in order to apply a greater force in a dynamic sports-specific skill.

## Figures and Tables

**Table 1:** Physical characteristics for male and female surfers.

	Male (n = 9)	Female (n = 8)
Age (yrs)	30.3 ± 7.3	25.5 ± 5.2
Height (cm)	176.4 ± 6.9 *	168.6 ± 3.8
Mass (kg)	76.2 ± 8.9 *	62.7 ± 7.6
Sum of 4 (mm)	35.1 ± 11.9 *	50.8 ± 16.7

* Significance at *p* ≤ 0.05.

**Table 2:** Reporting mean ± standard deviations of dynamic strength index (DSI) and dynamic skill deficit (DSD) for male and female surfers.

	Male n = 9	Female n = 8	*p*	*d*
Dynamic Strength Index (DSI)	0.79 ± 0.12	0.84 ± 0.08	0.33	0.48
Dynamic Skill Deficit (DSD)	0.72 ± 0.12	0.79 ± 0.02	0.32	0.69
